# The Cost-Effectiveness of Conservatively Managed Acute Appendicitis Versus Appendicectomy: A Systematic Review

**DOI:** 10.1097/SLE.0000000000001454

**Published:** 2026-03-24

**Authors:** Imran Farhad, Muhammad A. Khan, Aaron Kler, Mihir Trivedy, Dale Vimalachandran, Matthew Fok

**Affiliations:** * School of Medicine; † Institute of Systems, Molecular and Integrative Biology, University of Liverpool, Liverpool; ‡ The London School of Economics and Political Science (LSE), London; § Department of Colorectal Surgery, Countess of Chester Hospital, Chester, United Kingdom

**Keywords:** appendicitis, health economics, conservative treatment, appendicectomy

## Abstract

**Background:**

Appendicectomy remains the gold standard treatment for acute appendicitis. While there is continued debate as to the clinical benefit of conservative treatment in acute appendicitis, the cost-effectiveness of each treatment is a critical consideration. The aim of this study is to evaluate the current health economic evidence for surgery versus conservative management in acute appendicitis.

**Methods:**

A systematic literature search of 3 online databases for evidence pertaining to the economics of appendectomy compared with conservative management was performed according to PRISMA standards. The study protocol was prospectively registered with PROSPERO (study ID: CRD42023412691).

**Results:**

A total of 1219 patients were included (639 operative treatment vs. 580 conservative management). A total of 5 studies were included. Surgery was significantly more expensive when compared with conservative treatment (16.5% to 83.0%) percentage cost difference. The average length of hospitalisation was similar between the groups. The majority of studies reported a 12-month follow-up. The CHEERS assessment revealed a considerable risk of bias.

**Conclusion:**

Conservative management of appendicitis is the more economically effective treatment of the 2 modalities. This is an important consideration for resource-limited health care systems. However, further research with standardized methodologies and longer-term follow-up is warranted to fully assess the economic implications and clinical effectiveness of both treatment approaches.

**Key statement:**

Analysis indicates conservative management as the economically preferable option for acute appendicitis over surgery. This finding underscores its potential significance for resource-constrained health care systems. However, standardized studies with extended follow-up in different global health care settings are required to evaluate the economic and clinical merits of both treatment strategies.

Appendicectomy remains the gold standard treatment for acute appendicitis.^1^ However, there has been considerable interest in the conservative management of uncomplicated acute appendicitis with intravenous antibiotics. Previous systematic reviews of randomized controlled trials (RCTs) have been inconclusive on the benefit of the conservative treatment of appendicitis compared with appendectomy.^2,3^ A recent Cochrane review concluded that although conservative treatment is a feasible and safe treatment, nearly 15% of patients will fail conservative treatment and require surgery. The same review demonstrated that approximately a third of patients who were successfully treated with a conservative approach will require appendicectomy within 1 year.^4^ Conservative treatment can be attractive to patients as it avoids surgery, surgical scars, and potentially faster recovery.^5^ The pandemic of coronavirus disease 2019 (COVID-19), health systems and professional societies, were forced to reconsider the role of antibiotics for the conservative management of appendicitis. The Royal College of Surgeons in England recommended early in the COVID-19 pandemic that first-line treatment for acute uncomplicated appendicitis should be with antibiotics, citing fears over increased risk of death from COVID-19 following surgery and the possibility of transmission of COVID-19 from patient to theater personnel during surgery.^6^ Reattendance rate during the COVID-19 era for appendicitis-related issues was significantly higher, and a 1-year single-center follow-up study showed a 43.1% readmission rate for surgery.^6,7^

Surgery requires the dedicated expertise of at least 1 surgeon, an anesthetist, and a nurse, specialized equipment, anesthetic gases and drugs, a theater space, the ability to sterilize equipment, and a postoperative ward. This comes at a financial cost to health care systems and can be an important consideration in the delivery of services. However, there are also considerations that patients face with the prospect of surgery in countries where there is no universal health insurance, such as the United States, where up to 26 million people do not have any health insurance, or in low- and middle-income countries where resources, health care infrastructure, and finances are scarce.^8^

This systematic review is the first to assess and compare the health economics of surgery versus conservative treatment with antibiotics for acute appendicitis.

## METHODS

### Literature Search Strategy

**FIGURE 1:**
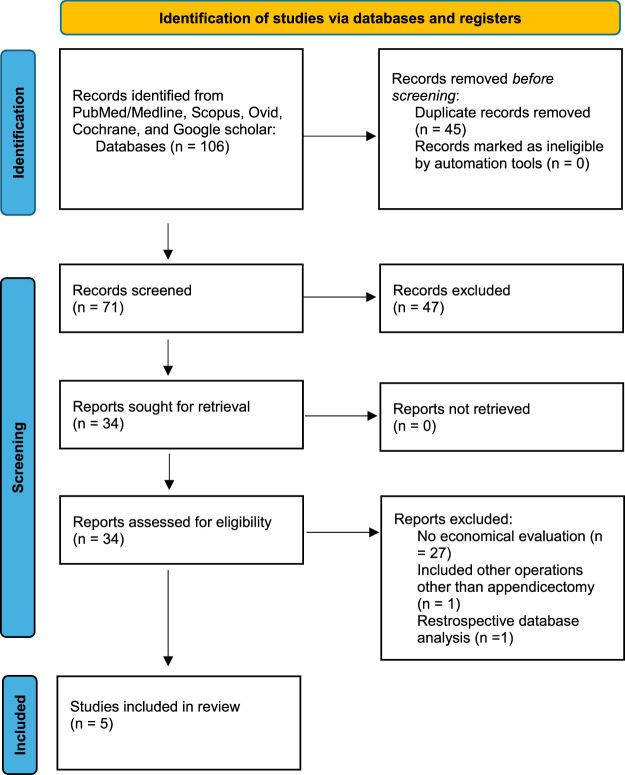
PRISMA diagram of included studies.

A comprehensive literature search was conducted according to the Preferred Reporting Items for Systematic Reviews and Meta-Analyses (PRISMA) guidelines. A search was performed on PubMed/Medline, Scopus, Ovid, Cochrane, and Google Scholar in January 2024. Search terms included: appendicectomy, appendectomy, laparoscopic surgery, laparotomy, antibiotic therapy, conservatively managed appendicitis, complicated appendicitis, uncomplicated appendicitis, perforated appendicitis, recurrent appendicitis, cost-benefit analysis, health economics, economic evaluation, watchful waiting, and active surveillance. All search terms were combined with Boolean operators and searched with MeSH terms to ensure maximal sensitivity. There were no limits on language. Titles and abstracts were screened using the inclusion criteria, and duplicates were excluded. Full-text article reference lists were searched for any further articles that were suitable for inclusion (Fig. 1). The search process was undertaken by 2 independent investigators (I.F. and M.A.K.), with discrepancies resolved by consensus with a third independent investigator (M.F.). This study was registered on the PROSPERO database (CRD42023412691) in July 2023, before conducting the final literature search. Costs were inflation-adjusted to January 2024 to align with the date of the final search and data extraction.

### Inclusion Criteria

Only prospective studies, including both randomized and nonrandomized studies, were included. Studies were included if they assessed surgery versus conservative treatment with antibiotics in acute appendicitis and also evaluated the economic cost of each treatment arm. We did not include pediatric studies, which represent a separate subtype of appendicitis.

### Methodological Quality Assessment of Included Studies

A qualitative assessment of bias of the included studies was performed using the revised tool for risk of bias (RoB-2).^9^ The quality of the included studies was rated by 2 reviewers, and discrepancies were resolved by consensus. The Consolidated Health Economic Evaluation Reporting Standards 2022 (CHEERS 2022) statement: updated reporting guidance for health economic evaluations was used to assess the quality and reporting standards of the economic evaluations in the included studies.^10^

The Consolidated Health Economic Evaluation Reporting Standards (CHEERS) tool is used to assess the quality with which economic evaluations are reported. It was developed by the International Society for Pharmacoeconomics and Outcomes Research (ISPOR) to promote more standardized and rigorous reporting of economic evaluations.^11^ The CHEERS tool includes a 24-item checklist and helps in interpreting and generalizability of studies.

### Data Extraction and Measured Outcomes

Data extraction was performed using a standardized proforma approved by 2 reviewers (I.F. and M.K.). The primary outcome was the cost of invasive treatment compared with the cost of conservative treatment (inflation-adjusted January 2024).^12^ The secondary outcomes included the number of patients who failed conservative treatment, the number of patients requiring surgery after discharge following conservative management, the mean length of hospital stay of both groups, and the median follow-up.

### Statistical Analysis

The planned synthesis of data included standard descriptive statistics (reported as means with 95% CIs or median with interquartile range, where appropriate) to summarize the demographic and operative data of recruited patients across all eligible studies. Meta-analysis was not planned due to the heterogenous of the primary outcome.

## RESULTS

The literature search identified 71 potential studies. Subsequent to screening of the titles and abstracts, 66 papers were excluded. Five studies for which the full text was obtained and reviewed.^13–17^ Of these papers, the reference lists were searched to look for any potential papers to include, but this did not reveal any further papers. A total of 5 studies met the inclusion criteria for the study^13–17^ (Fig. 1). This included a total of 1219 patients. There was a predominance of males within the cohort of patients, which was consistent across all studies (Table 1). The majority of studies were performed in Western countries. Two studies were performed in the United States,^15,17^ 3 in Europe (1 in Sweden,^14^ 1 in Finland),^13^ and 1 in Turkey.^16^ Three studies reported a 1-year follow-up,^14,15^ and 1 study reported an average follow-up of 19.91 months.^16^ Mean age and male:female ratios were similar between groups. Importantly, there were more patients who underwent open appendicectomy compared with laparoscopic appendicectomy (n=296 vs. 174, respectively). However, Hannson et al^14^ did not report the number of patients who had open versus laparoscopic surgery for their cohort of 369 patients.

**TABLE 1:** Table of All Included Studies and Their Respective Patient Cohort Characteristics

References	Year	Country	Total patients (n)	Operative group (n)	Conservative group (n)	Average age in the operative group	Average age in the conservative group	Male:female ratio in the operative group	Male:female ratio in the conservative group	Open appendicectomy (n)	Laparoscopic appendicectomy (n)
Turhan et al^16^	2009	Turkey	290	183	107	26.25±0.79	30.98±1.30	125:58	65:42	33	150
Sippola et al^18^	2017	Finland	530	273	257	(Range: 18-60)	(Range: 18-60)	NR	NR	258	15
Talan et al^15^	2017	USA	30	16	14	(Median: 36)	(Median: 31)	9:5	9:7	5	9
Hansson et al^14^	2009	Sweden	369	167	202	38±1.00	38±1.00	92:75	103:99	NR	NR
Wu et al^17^	2015	USA	NR	NR	NR	NR (18+)	NR (18+)	NR	NR	0	All

The cost of treatments in all studies has been converted to US dollars and adjusted for inflation in January 2024. In all studies, conservative treatment of appendicitis with antibiotics was significantly cheaper (Table 2). This was particularly true of the 2 studies performed in the United States (surgery $15,805.41 and $15,697.57 for surgical treatment vs. $6533.21 and $13,300.46 for conservative management; Table 2).^15,17^ The percentage difference in cost between conservative and surgically treated patients ranged from 16.5% to 83%. Surgery was significantly cheaper in countries outside of the United States. The study performed in Turkey (Turhan et al^16^) stands as an outlier in that the average cost of both surgical and conservative treatment was significantly lower compared with all others ($796.47 for surgery and $616.95 for conservative treatment).^16^ The 2 studies performed in Sweden and Finland produced similar results for surgical and conservative treatment ($4041.57 and $4848.05 for surgical treatment vs. $2533.48 and $3502.85 for conservative treatment).^13,14^

**TABLE 2:** Table Describing the Economic Analysis Performed in Each Study

References	Average cost of operation (native currency)	Average cost of conservative treatment (native currency)	Cost of operation (inflation-adjusted January 2024)	Cost of conservative treatment (inflation-adjusted January 2024)	Percentage difference (%)	Average follow-up (months)	Patients who needed surgery after conservative treatment (n)	Average length of hospital stay for conservative group (mean±SD)	Average length of hospital stay for operative group (mean±SD)
Turhan et al^16^	755 TL	585 TL	$796.47	$616.95	25.4	19.91±0.35 (in conservative group)	7	3.14±0.10 d	2.4±0.14 d
Sippola et al^18^	€2882.00	€1806.60	$4041.57	$2533.48	45.9	1 y	15	3.2±0.9 d	2.8±1.0 d
Talan et al^15^	$12447 (median)	$5145 (median)	$15805.41	$6533.21	83.0	1 y	1	0.68 d	1.75 d
Hansson et al^14^	SEK 36400.00	SEK 26300.00	$4848.05	$3502.85	32.2	NR (1 mo and 12 mo follow ups)	11	3.0±0.1 d	3.0±0.3 d
Wu et al^17^	$12213	$10348	$15697.57	$13300.46	16.5	NR	NR	NR	NR

€ indicates Euro; NR, not recorded; SEK, Swedish Krona; TR, Turkish Lira; $, US Dollar.

A breakdown of the costs, including complication costs, was described by Hannson et al, which included materials, medical drugs, radiology and surgery resources, postoperative surveillance, laboratory tests, and pathology for both groups. Wu et al included perioperative complication costs in patients who underwent appendectomy, but did not specify whether complication costs included antibiotic therapy, and also assumed outpatient follow-up after hospitalization was equivalent in both groups. Turhan et al included nonoperative costs, recurrent admissions without surgery, in their economic analysis, and included surgical complications.

The average length of stay was variable between each group and ranged between 1 and 3 days. Average follow-up is important to capture the number of patients who undergo conservative treatment who later require surgery for recurrent appendicitis, and consequently, is incorporated into the cost. The longest follow-up was seen in the multicentre Finnish trial, which included patients up to 5 years.^13^

**FIGURE 2:**
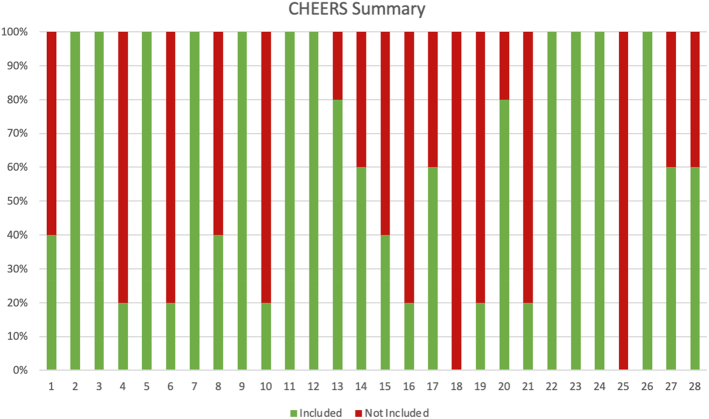
Bar graph format for the Consolidated Health Economic Evaluation Reporting Standards (CHEERS) for the included studies.

In this study, the CHEERS tool identified that reporting of the methodological quality and results was inadequately reported in the majority of studies (Table 3 and Fig. 2). In particular, there were 2 domains (characterizing heterogeneity and the effect of engagement with patients and others affected by the study) that were not reported in any studies. The ROBINS-I and RoB-2 tools identified that the risk of bias from the studies was high (Supplemental Fig. 1, Supplemental Digital Content 1, http://links.lww.com/SLE/A529; Supplemental Fig. 2, Supplemental Digital Content 2, http://links.lww.com/SLE/A530).

**TABLE 3:** Consolidated Health Economic Evaluation Reporting Standards (CHEERS) For the Included Studies

Section	Item description	Item	Included (%)	Not included (%)
Title	Title	1	40	60
Abstract	Abstract	2	100	0
Introduction	Background and objective	3	100	0
Methods	Health economic analysis plan	4	20	80
Methods	Study population	5	100	0
Methods	Setting and location	6	20	80
Methods	Comparators	7	100	0
Methods	Perspective	8	40	60
Methods	Time horizon	9	100	0
Methods	Discount rate	10	20	80
Methods	Selection of outcomes	11	100	0
Methods	Measurement of outcomes	12	100	0
Methods	Valuation of outcomes	13	80	20
Methods	Measurement and valuation of resources and costs	14	60	40
Methods	Currency, price date, and conversion	15	40	60
Methods	Rationale and description of the model	16	20	80
Methods	Analytics and assumptions	17	60	40
Methods	Characterizing heterogeneity	18	0	100
Methods	Characterizing distributional effects	19	20	80
Methods	Characterizing uncertainty	20	80	20
Methods	Approach to engagement with patients and others affected by the study	21	20	80
Results	Study parameters	22	100	0
Results	Summary of main results	23	100	0
Results	Effect of uncertainty	24	100	0
Results	Effect of engagement with patients and others affected by the study	25	0	100
Discussions	Study findings, limitations, generalizability, and current knowledge	26	100	0
Other relevant	Source of funding	27	60	40
Other relevant	Conflicts of interest	28	60	40

## DISCUSSION

This systematic review suggests that conservatively treated appendicitis is a cheaper alternative than surgically treated appendicitis. In each included study, conservative treatment consistently demonstrated lower costs when compared with surgical methods. There was a large cost disparity in the United States compared with all other countries, where both conservative and surgical treatments were substantially higher.^19^ The care provided for patients in the United States is comparatively similar to that of other countries with similar health care provisions.^19^ There is evidence to suggest that the reason why costs are significantly higher in the United States is because of a for-profit system that relies on the prices charged by hospitals, insurance companies, drug and device companies. In addition, administrative costs, higher salaries for medical professionals, and the frequent use of advanced diagnostic procedures also contribute to the inflated health care costs in the United States compared with other high-income nations.^20^ These financial factors make the conservative management of appendicitis with antibiotics an important consideration in the United States, where patients without comprehensive insurance coverage may disproportionally face significant out-of-pocket expenses for surgical treatment.^21^

Appendicitis is one of the most common emergency surgical conditions worldwide.^22^ Laparoscopic appendicectomy remains the most common treatment for patients presenting with acute appendicitis.^23^ However, there has been a great deal of interest in the conservative approach with antibiotic treatment, and its clinical utility is still debated. While clinical outcomes have been the primary focus of many studies, economic considerations are equally important when evaluating treatment strategies. Despite this, the cost-effectiveness of nonoperative management has not previously been systematically reviewed. A comprehensive analysis of the economic impact is essential to inform clinical decision-making and health care policy, particularly as health care systems seek more sustainable models of care.

Low- and middle-income countries (LMICs) represent a difficult challenge in health care, but also in surgery. It is well acknowledged that ∼90% of patients in LMICs lack access to timely surgical care.^24,25^ A recent scoping review of 78 studies identified that one of the main factors delaying the receipt of surgical care for appendicitis was financial concerns.^26^ Furthermore, lack of adequate transport and infrastructure, including financial challenges for transport to a medical facility with surgical facilities, was identified as another significant factor to delay the treatment of appendicitis. Therefore, this study highlights that there is potential in treating patients conservatively with antibiotics as an alternative to surgery in select cases of uncomplicated appendicitis, particularly in LMICs. Conservative management could help mitigate the financial burden, address logistical barriers, and offer a viable solution where access to surgical facilities is limited. However, the effectiveness and safety of this approach must be carefully weighed, as delayed surgical treatment can lead to a higher mortality rate.^27,28^ There is selective evidence to suggest that faecoliths are a risk factor for failure of conservative treatment.^29,30^ However, identifying these patients requires access to computer tomography (CT) scans, which in countries where resources are limited, and patients are already suffering with financial worries, may not be a viable strategy for patient selection. The development of context-specific treatment protocols, investment in infrastructure, and improvement of health care financing systems are critical to addressing the unmet surgical needs in these regions.

The largest study to evaluate the cost-effectiveness of these approaches is the multicenter Finnish APPAC study.^13^ This study recruited patients with uncomplicated appendicitis without evidence of a faecolith as determined by CT scan. Despite the trial being published in 2015, a major limitation is that a high number of appendicectomies were performed open. Open appendicectomy is known to have an increased complication rate compared with laparoscopic surgery and, consequently, may increase the costs.^31^ Furthermore, in the surgical group, patients were required to have a 3-day length of stay, which would also affect costs. The predominance of open procedures in this trial is a limitation, especially considering that laparoscopic appendicectomy is now the gold standard. While open appendicectomy is often perceived as less expensive due to the avoidance of disposable instruments and the need for specialized equipment such as tower stackers and insufflation systems, recent evidence suggests that the cost difference between open and laparoscopic techniques is minimal.^32,33^

This study is limited by the heterogeneous nature of studies that have been included, as identified with the CHEERS, RoB-2, and ROBIN-I tools. In this study, the majority of appendicectomies were performed open. The laparoscopic approach is the preferred and standard approach to appendicectomy, and this makes comparison difficult with current practice. The open approach may increase the number of complications, which will increase the cost of the intervention. However, the laparoscopic approach requires significantly more and costlier equipment, which can also affect the cost. Although a strength of this study is that the studies included were across different countries, it is also a weakness, considering the variability of costs reflecting the complexity of different countries’ health care systems and global health economics. Finally, only 1 study was based in an LMIC,^16^ and this makes it difficult to extrapolate meaningful conclusions about the cost-effectiveness of treatments in acute appendicitis across different countries.

In conclusion, this systematic review suggests that conservative treatment of appendicitis using antibiotics presents a potentially cost-effective alternative to surgical intervention, particularly in countries like the United States, where health care costs are significantly higher. However, while conservative treatment offers potential cost savings, it is not without its challenges, particularly in the context of low- and middle-income countries (LMICs) where access to diagnostic tools like CT scans and timely surgical care is limited. At present, there is no reliable predictive tool for response to conservative treatment in acute appendicitis. In parallel to researching the right patient selection for conservative treatment, future research should focus on assessing the long-term outcomes of conservative versus surgical treatment of appendicitis in various health care settings, with particular emphasis on LMICs. Developing standardized treatment protocols and improving health care infrastructure will be key to addressing the global burden of appendicitis, ensuring that both cost and patient outcomes are optimized across the globe.

## Supplementary Material
