# Supplementary figures and images for: The Cost-Effectiveness of Conservatively Managed Acute Appendicitis Versus Appendicectomy: A Systematic Review
Source: Surg Laparosc Endosc Percutan Tech. 2026 Mar 24;36(3):e1454. doi: 10.1097/SLE.0000000000001454 (PMC13225105; doi:10.1097/SLE.0000000000001454)

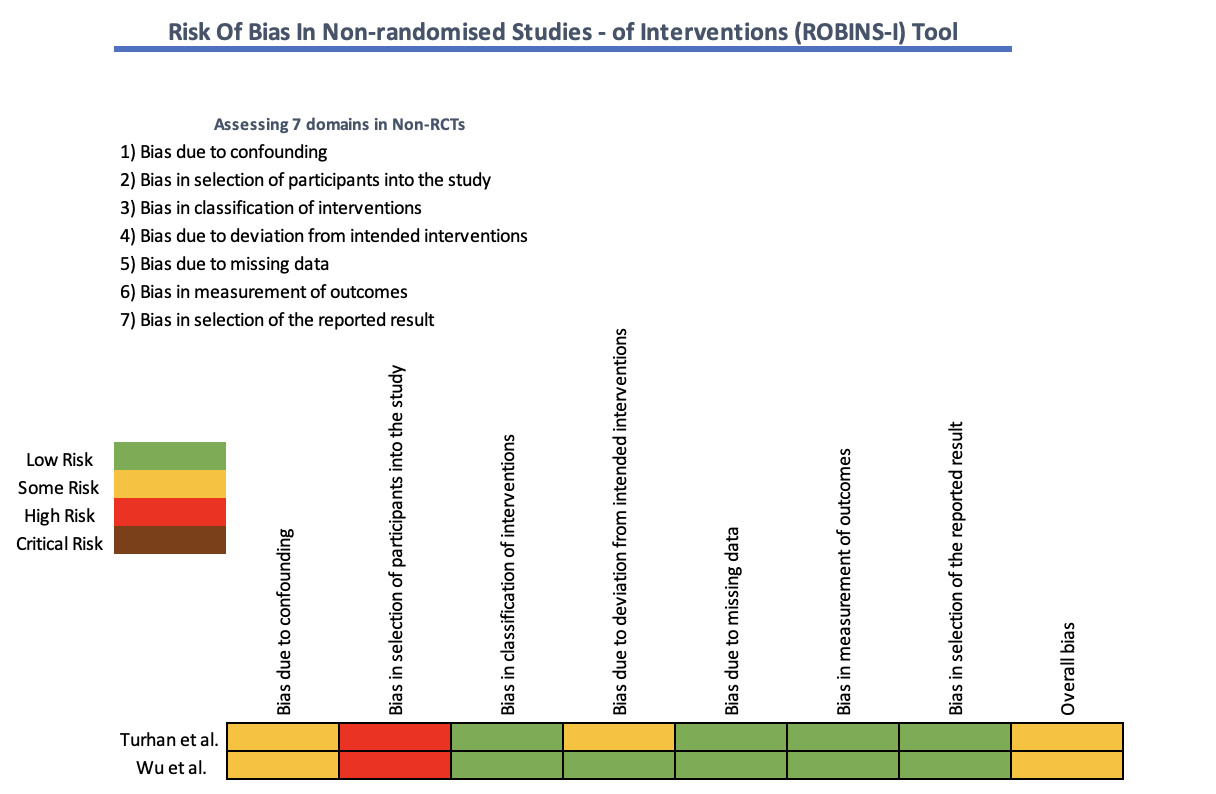


Supplementary Figure 1.

Supplement: Supplementary file 1 [file sle-36-e1454-s001.docx]

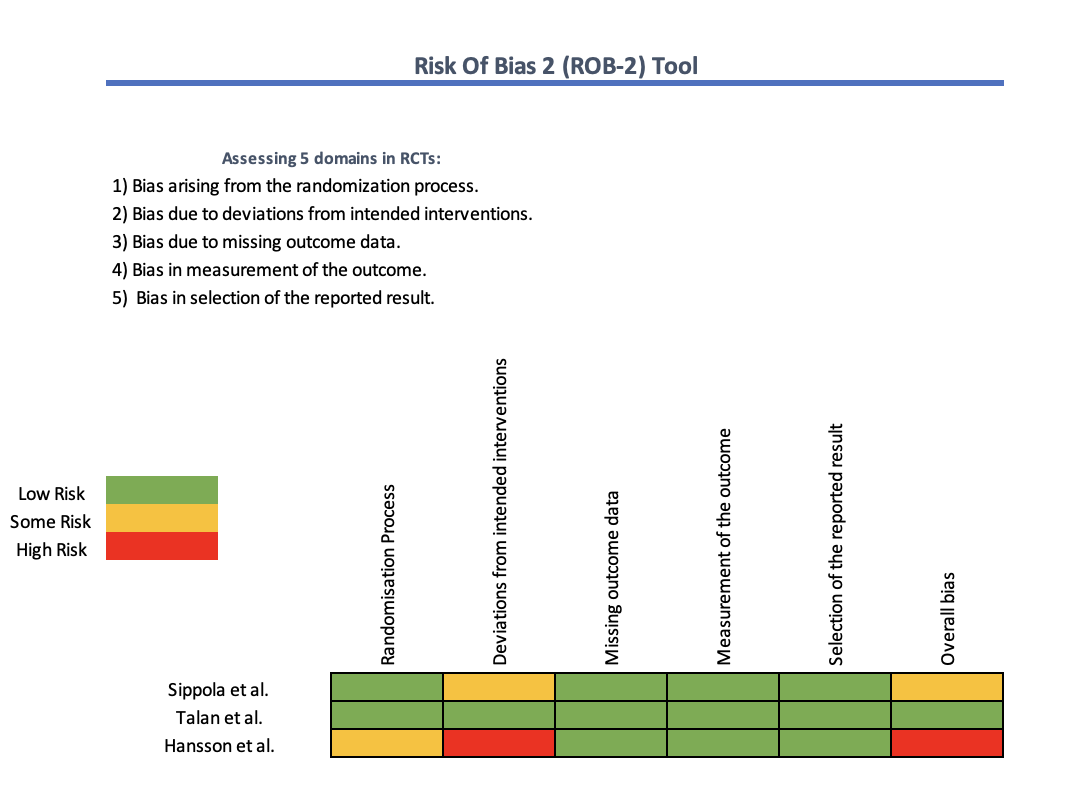


Supplementary Figure 2.

Supplement: Supplementary file 2 [file sle-36-e1454-s002.docx]
